# Young cerebrospinal fluid contains key rejuvenating factors

**DOI:** 10.1093/lifemeta/loac005

**Published:** 2022-06-03

**Authors:** Dongming Zhang, Dongsheng Cai

**Affiliations:** Department of Molecular Pharmacology, Albert Einstein College of Medicine, Bronx, NY, USA; Department of Molecular Pharmacology, Albert Einstein College of Medicine, Bronx, NY, USA

**In a recent study published in *Nature*, Iram *et al*. reported that treatment with young cerebrospinal fluid can play a rejuvenating function in older mice by promoting the proliferation and differentiation of oligodendrocyte progenitor cells in the hippocampus for cognitive improvement.**

It was shown more than a decade ago that replacing the blood of old mice with blood from young mice can improve tissue functions and reverse some aging-associated phenotypes [1]. This result has sparked a flurry of research, including the studies showing that young mice-derived blood or human umbilical cord plasma proteins can support or promote learning and memory in aged mice [2,3]. Brain aging has been considered to predispose to neurodegenerative diseases; however, the brain is mainly isolated from the blood circulation due to the blood–brain barrier that limits the use of these methods for targeting aging brain. The cerebrospinal fluid (CSF) is essential and immediate environment for the brain, which serves to not only deliver nutrients but also provide a platform for various types of brain cells to communicate with each other. The protein composition in human CSF is known to be altered with aging. For example, there is a decrease in CSF concentration of brain-derived neurotrophic factor in older individuals than in young controls [4]. Recently, it was consistently demonstrated that hypothalamic cells, including stem/progenitor cells and neurons, are important for secreting factors such as microRNAs as well as proteins like parathymosin into the CSF, while age-dependent declines of these functions are closely related to the central control of aging [5–8]. Thus, it is highly required to directly examine whether young CSF could protect against aging-associated disorders, in particular neurological disorders.

In a recent study published in *Nature*, Iram *et al*. found that treatment with CSF from young mice plays a rejuvenating function in older mice [9]. The researchers collected CSF from young mice at the age of 10 weeks and injected the fluid into the lateral ventricle of the brain in older mice at the age of 18 months. To minimize the force of injection, which might increase the pressure around the brain, a pump was implanted into the brains of older mice, which allowed to slowly pump the CSF into the brains of mice. Three weeks later, the mice were tested with remote fear memory, the result showing that the mice treated with young mice-derived CSF significantly improved memory function than those treated with artificial CSF. Subsequently, the authors employed bulk RNA-seq to analyze the molecular targets of young mice-derived CSF in the hippocampus at the transcriptional level. Results showed that oligodendrocytes were the most responsive to the treatment of young mice-derived CSF. Through labeling assays, the authors found that CSF from young conditions can stimulate proliferation and differentiation of oligodendrocyte progenitor cells (OPCs) in the hippocampus of aged mice. In addition, it was found that the levels of some protein markers and the number of myelinated axons in the hippocampus increased upon the treatment with young mice-derived CSF. These results supported that the CSF of young mice contains essential factors for improving cognition, which is associated with oligodendrocyte regeneration in the hippocampus.

The authors further investigated the potential molecules in CSF that are essential for promoting the regeneration of oligodendrocytes. By using SLAMseq, researchers detected newly synthesized RNA transcripts in rat OPCs in culture after exposure to young human CSF. Remarkably, serum response factor (Srf) was found to be significantly induced upon the treatment with CSF from young mice. Using Srf-knockout OPCs, the authors confirmed that SRF is indispensable for young CSF-induced OPCs proliferation. Consistent with this result, the authors further demonstrated that SRF expression in OPCs in the hippocampus decreased with age, while SRF pathways were activated in old mice after administrating CSF from young mice. Next, the researchers analyzed the molecules and growth factors in the CSF that are crucial for SRF activation. Based on the available protein databases, a list of candidates were screened through a reporter cell model, the result revealing that Fibroblast growth factor 8 (Fgf8) and Fgf17 strongly activated the SRF signaling pathway. The authors focused on Fgf17, which is known to be highly expressed in the brain, and found that Fgf17 induced OPC proliferation in cultured rat OPCs. Furthermore, Fgfr3 seemed essential for the action of Fgf17 in activating SRF pathway. Finally, the researchers infused Fgf17 into the brains of old mice, the result showing that Fgf17 was indeed able to induce OPC proliferation and related improvement in long-term memory. Further treatment with Fgf17 blocking antibody led to impairments in cognitive function of young mice. Of interest, the authors showed that neurons are significant for producing Fgf17, in line with a recent study showing that brain neurons generally bear endocrine functions [8].

To summarize, this study revealed that the CSF is important for hippocampal oligodendrocyte regeneration and protection against aging-related cognitive decline. Overall, the study provides convincing evidence to support the idea that young CSF contains key rejuvenating factors, among which Fgf17 is a critical protein factor for OPC regeneration. Based on this finding other factors in young CSF that are important for brain health and for anti-aging potentials need to be further identified. Besides oligodendrocytes and hippocampus, the anti-aging functions of young CSF probably involve various brain cell types as well as various brain regions, which might even possibly include some peripheral tissues as targets of the CSF [5–7]. Moreover, in addition to peptides and proteins, other secretory factors in the CSF such as small RNA species could play important roles [5–7], and it should be informative to study if these nonprotein factors could be engaged with the effects of Fgf17 in an interactive or independent manner. Taken together, identifying CSF factors in promoting rejuvenation represents a critical research direction in the areas of addressing aging biology and medicine.
